# RETRACTED: Masataka, N. Possible Anxiolytic Effects of Cannabidiol (CBD) Administration on Feline Responses to a Fear Response Test. *Animals* 2025, *15*, 1642

**DOI:** 10.3390/ani16121817

**Published:** 2026-06-12

**Authors:** Nobuo Masataka

**Affiliations:** Center for Research of Developmental Disorders, Kyoto 612-0046, Japan; masa5taka5@gmail.com

The journal retracts the article titled “Possible Anxiolytic Effects of Cannabidiol (CBD) Administration on Feline Responses to a Fear Response Test” [1], cited above.

Following publication, concerns were brought to the attention of the Editorial Office regarding the degree to which this study adheres to MDPI’s policies on Research Involving the use of animals.

In accordance with standard journal procedures, the Editorial Office and Editorial Board conducted an investigation that confirmed the study did not obtain valid ethical approval and is not compliant with MDPI policy for research involving the use of animals.

As a result, the Editorial Board has decided to retract this paper as per MDPI’s retraction policy.

This retraction was approved by the Editor-in-Chief of the journal *Animals*.

The authors have been informed of this retraction but did not provide a comment on this decision.
